# CAR-based cell therapy for autoimmune diseases

**DOI:** 10.3389/fimmu.2025.1613622

**Published:** 2025-09-18

**Authors:** Xiu Li, Chao He, Ke Wang, Guo-Ying Xu, Ya-Chao Xie, Xin-Yu Ying

**Affiliations:** ^1^ Central Laboratory, Department of Clinical Laboratory, Fourth Affiliated Hospital of Jiangsu University, Zhenjiang, Jiangsu, China; ^2^ Institute of Medical Genetics and Reproductive Immunity, School of Medical Science and Laboratory Medicine, Jiangsu College of Nursing, Huaian, Jiangsu, China; ^3^ Department of Clinical Laboratory, Ningbo Medical Center Lihuili Hospital, Affiliated Hospital of Ningbo University, Ningbo, Zhejiang, China

**Keywords:** chimeric antigen receptor, autoimmune disease, cell therapy, synthetic biology, CAR-T cell

## Abstract

Chimeric antigen receptor (CAR)-based cell therapies, initially designed for oncology, are rapidly advancing as a novel and highly targeted approach for the treatment of autoimmune diseases (AIDs). By harnessing engineered immune cells to eliminate autoreactive immune components or restore immune homeostasis, CAR-based strategies offer new avenues beyond conventional immunosuppression. In this review, we summarize current applications of CAR-T cells in autoimmune diseases, and discuss emerging approaches including CAR-Tregs, chimeric autoantibody receptor T (CAAR-T) cells, CAR-NK cells, and CAR-macrophages. We also describe advances in CAR design, including antigen selection, co-stimulatory domains, and safety control mechanisms, which are critical for improving therapeutic precision and reducing side effects. In addition, we highlight the role of synthetic biology in enabling more flexible and controllable CAR functions. Finally, we discuss the main challenges facing clinical translation, such as antigen specificity, long-term persistence, and manufacturing feasibility. These developments collectively support the potential of CAR-based therapies as a next-generation option for autoimmune disease treatment.

## Introduction

Autoimmune diseases (AIDs) represent a heterogeneous group of disorders characterized by impaired self/non-self-recognition, leading to immune system dysregulation and subsequent attacks on the body’s own tissues. These autoimmune responses involve the activation of autoreactive lymphocyte clones and the production of autoantibodies against self-antigens, resulting in immune-mediated damage that affects multiple organs. Notably, autoreactive B-cell clones and autoantibodies targeting self-antigens are present even before the onset of clinical symptoms (1).

AIDs exhibit significant complexity in both their underlying mechanisms and clinical manifestations, ranging from mild laboratory abnormalities to life-threatening acute organ failure. This heterogeneity poses substantial challenges in clinical management and therapeutic development. Currently, the primary therapeutic approach for AIDs relies on broad-spectrum immunosuppressants and neutralizing antibodies. While these agents can effectively control disease progression, complete remission is rarely achieved. For instance, although autoreactive B-cells play a critical role in autoantibody production in most AIDs, targeting them with monoclonal antibodies such as Rituximab and Inebilizumab has demonstrated only limited efficacy, largely due to the persistence of these cells in lymphoid organs and affected tissues (2).

Chimeric antigen receptor (CAR)-based cell therapies—originally developed for B-cell malignancies, are now being repurposed for AIDs treatment. Specifically, autologous CAR-T cells targeting CD19 have shown rapid and sustained depletion of circulating autoreactive B-cells, leading to clinical and serological remission in RA (3). However, CAR-T therapy in patients with autoimmune diseases is not without adverse effects, most commonly presenting as transient B-cell aplasia and low-grade CRS. Importantly, the risks of lymphodepletion-induced immunosuppression and infectious complications appear to be markedly reduced in AIDs compared to oncologic contexts, likely due to the preservation of hematopoietic niches and accelerated immune reconstitution in non-malignant conditions (4). These risks raise concerns about the therapeutic index of CAR-T cells in non-malignant settings. To mitigate these issues, engineered CAR-T cells incorporating “safety switches” and immunomodulatory elements have been developed to allow for controlled activation, function, and persistence (5).

Recent studies have elucidated key characteristics of AIDs that distinguish them from cancers, including the pathological role of autoantibodies, the need for immune homeostasis reconstitution in solid tissues, and the poor quality of patient-derived immune cells. Such factors may limit the effectiveness of conventional CAR-T approaches. In response, researchers have pursued more tailored strategies, including *in vivo* gene delivery approaches to bypass the need for preconditioning lymphodepletion, the use of mRNA-based CARs for transient expression, and the design of chimeric autoantibody receptor T cells (CAAR-T) that express autoantigen epitopes in place of conventional scFvs to selectively eliminate autoreactive B-cell clones (6, 7). CAR-Tregs generated from CD4^+^ T cells are also being investigated to re-establish immune tolerance in affected tissues (8). Given the prolonged manufacturing timelines and substantial commercial costs of autologous CAR-T cells for AIDs treatment, allogeneic immune cells are being explored for the development of “off-the-shelf” CAR-T products (9). Additionally, CAR-macrophages have been designed for improved tissue infiltration, and CAR-NK, which are not restricted by major histocompatibility complex (MHC), offer potential for allogeneic application.

Beyond expanding cell platforms and refining CAR designs, advances in synthetic biology have introduced new possibilities for CAR-based therapies. For instance, the “OR-gate” design–such as CD19/BCMA bispecific CAR-T cells–enables targeting of either CD19^+^ B-cells or BCMA^+^ plasma cells, thereby enhancing therapeutic breadth (10). In contrast, “AND-gate” design requires dual-antigen recognition to activate the cell, thereby improving specificity and reducing off-target effects. In addition, the synthetic Notch (synNotch) system, a modified version of Notch signaling pathway, enables signal-dependent gene transcription by releasing natural or synthetic transcription factors upon antigen engagement (11).

In this review, we discuss recent advancements in CAR-based therapies for AIDs, focusing on improving efficacy and safety, selecting suitable cell platforms and CAR designs tailored to disease-specific characteristics, and leveraging synthetic biology to create innovative therapeutic strategies.

## Current treatments of autoimmune disease

AIDs primarily result from inflammatory responses, cytolysis, and immune complex deposition, driven by the activation of autoreactive T and B-cells that continuously release excessive inflammatory factors, leading to tissue damage. Consequently, anti-inflammatory therapies have become a primary choice in AIDs treatment (12). Steroidal anti-inflammatory drugs (SAIDs), such as dexamethasone and prednisone, are widely used to inhibit the activation and infiltration of autoreactive T cells by suppressing prostaglandins and leukotrienes production. Although these hormonal treatments exhibit significant short-term efficacy, their long-term efficacy is limited by frequent disease relapse upon discontinuation and the emergence of adverse effects associated with chronic immunosuppression, including increased susceptibility to infections, hypertension, and osteoporosis (13). In addition to steroids, nonsteroidal anti-inflammatory drugs (NSAIDs), such as aspirin and diclofenac, are commonly employed to alleviate localized joint inflammation in patients with RA. However, NSAIDs lack immunomodulatory properties and are ineffective in controlling the underlying disease progression. Moreover, prolonged NSAID use is associated with a wide range of adverse effects, including central nervous system abnormalities, cardiovascular complications, gastrointestinal disturbances, hematological alterations, and hepato-renal dysfunction (14). Given these limitations, concerns remain regarding the efficacy and safety of anti-inflammatory drugs in AIDs management.

To address these challenges, small-molecule drugs and neutralizing antibodies/receptors have become the alternative therapeutic strategies for AIDs. The Janus kinase-signal transducer and activator of transcription (JAK-STAT) signaling pathway plays a crucial role in mediating inflammatory cytokines production, including interleukin-2 (IL-2) and IL-6, and tumor necrosis factor-alpha (TNF-α), all of which contribute to AIDs pathogenesis (15). Tofacitinib, a JAK inhibitor, has been clinically validated to relieve inflammation responses in AIDs by the inhibition of JAK1 and JAK3. However, the secretion of some key pro-inflammatory factors, including TNF-α, IL-1 and IL-17, is independent from the JAK pathway, necessitating the exploration of alternative therapeutic approaches (16). For patients exhibiting excessive TNF-α production who are resistant to Tofacitinib, neutralizing antibodies or soluble TNF-α receptors can serve as “traps” to neutralize and sequester free TNF-α, thereby mitigating tissue injury and inflammation. Beyond TNF-α, common neutralizing targets in AIDs include IL-1(targeted by Anakinra), IL-6 (targeted by Tocilizumab) and IL-17 (targeted by Secukinumab) (17–19). While these neutralizing antibodies provide effective symptomatic relief, they do not directly target the underlying immune dysregulation, such as the breakdown of immune tolerance and the persistent activation of autoreactive T and B-cells. IL-2, a pleiotropic cytokine, exhibits context-dependent immune regulatory functions (20). In the context of AIDs, CD4^+^ regulatory T cells (Tregs) prevent lethal autoimmunity in IL-2 receptor β-deficient mice, highlighting the critical role of IL-2 in Tregs-mediated immunosuppression (21). Subsequent studies revealed that IL-2 signaling through its high-affinity receptor, CD25 enhances Tregs function by promoting Forkhead box P3 (FoxP3) expression via the JAK3-STAT5 pathway (22). A 2016 clinical trial evaluating low-dose IL-2 therapy for SLE demonstrated selective modulation of Tregs, follicular helper T cells (Tfh), and IL-17-producing helper T cells (Th17) (23). To date, low-dose IL-2 therapy has achieved clinical remission in AIDs conditions such as primary Sjögre’n Syndrome and SLE with minimal adverse effects (24, 25).

Despite these promising findings, IL-2 therapy faces challenges, including a short half-life requiring frequent dosing and limited efficacy in suppressing autoantibody production (26). Given the essential roles of B-cells in autoantibody production, targeting B-cells remains a viable therapeutic strategy. Rituximab, a CD20-specific monoclonal antibody approved in 2004, facilitates B-cell depletion primarily through antibody-dependent cellular cytotoxicity (ADCC) and has demonstrated clinical benefits in RA. However, its broader application in other AIDs, such as SLE, has been constrained by the incomplete elimination of autoreactive B-cell reservoirs within lymphoid tissues (27). To address this limitation, Obinutuzumab—a humanized, glycoengineered anti-CD20 antibody which incorporates modifications that augment FcγRIII binding affinity—was developed with enhanced affinity for FcγRIII, resulting in stronger ADCC activity while attenuating complement-dependent cytotoxicity (28). In a phase II trial (NCT02550652) evaluating Obinutuzumab for the treatment of proliferative lupus nephritis, patients receiving standard therapy (mycophenolate and corticosteroids) combined with Obinutuzumab achieved a significantly higher complete renal response (CRR) rate (41%) compared to those receiving standard therapy alone (23%) at 104 weeks post-treatment (29). Another B-cell-targeting strategy involves inhibition of B cell survival and activation. Belimumab, a fully human recombinant IgG1 kappa monoclonal antibody that neutralizes B-cell-activating factor (BAFF), has demonstrated significant efficacy in reducing autoantibody titers and flare frequency, earning regulatory approval for the treatment of SLE in 2011 (30). Nevertheless, the need for repeated dosing and associated adverse effects, such as decreased IgG levels and heightened susceptibility to infections, have limited its widespread adoption (31).

Current therapies for AIDs primarily aim to inhibit inflammatory responses and restore immune tolerance. Although some treatments have achieved temporary clinical success in specific AIDs subtypes, they are often limited by immunosuppression-associated infection, incomplete depletion of autoreactive cell, and the requirement for repeat dosing. Moreover, inter-patient heterogeneity and the complex nature of these diseases mean that a substantial proportion of patients fail to respond adequately to existing therapies (32). Therefore, there remains an urgent need for a universal therapeutic approach that capable exerting precise immune modulation across multiple AIDs, while maintaining an optimal balance between therapeutic efficacy and safety.

## CAR-T therapies in tumor and autoimmune diseases

Over the past decades, treatments for B-cell-driven malignancies have significantly benefitted from CAR-based strategies. CD19-targeted CAR-T cells, which induce potent B-cells depletion, have achieved remarkable success in clinical practice (33–35). The standard procedure involves isolating T cells from the patient’s peripheral blood, transducing them with a CAR construct specific to CD19, expanding the modified T cells *in vitro*, and reinfusing them into patients following lymphodepletion (36, 37).

**Figure 1:**
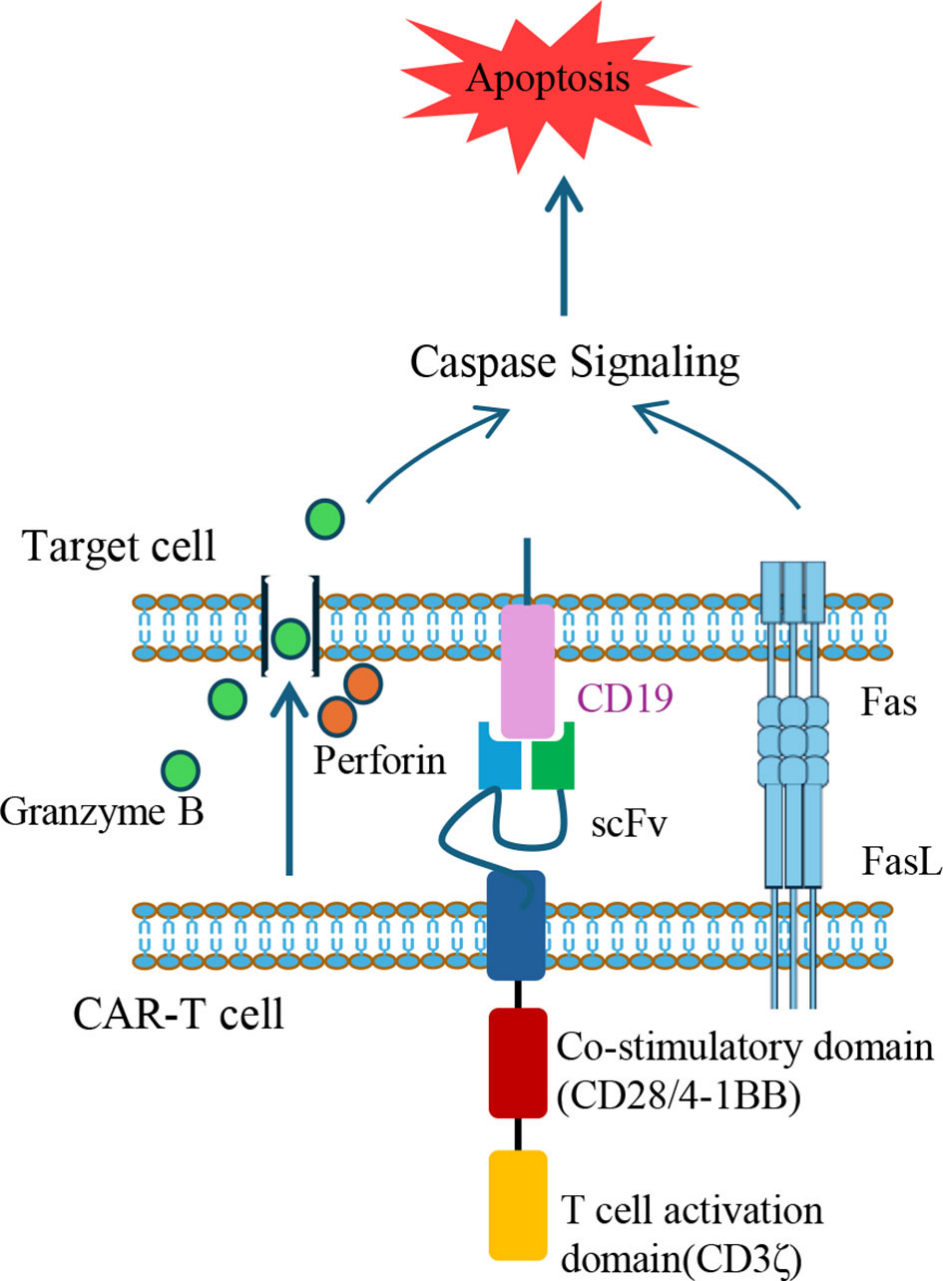
Mechanisms of target cell apoptosis induced by CAR-T cells. CAR-T cells eliminate target cells through two primary apoptotic pathways. One involves the secretion of perforin to create pores in the target cell membrane, allowing granzyme B to enter and activate caspase cascades. The second mechanism involves FasL expression on CAR-T cells, which engages Fas on target cells, triggering Caspase-dependent apoptotic signaling. The illustrated CAR construct contains a scFv targeting CD19, a co-stimulatory domain (e.g., CD28 or 4-1BB), and a T cell activation domain (CD3ζ). FasL, Fas ligand; scFv, single-chain variable fragment; CAR, chimeric antigen receptor.

The fundamental structure of CARs typically comprises three components: an extracellular single-chain variable fragment (scFv) that recognizes the target antigen, a transmembrane domain, and an intracellular signaling domain. In therapies for B-cell malignancies such as non-Hodgkin lymphoma or B-acute lymphoblastic leukemia (B-ALL), the scFv are typically designed to target CD19, a surface marker consistently expressed throughout B-cell development (38). The intracellular domain incorporates the CD3ζ signaling motif of the T-cell receptor (TCR)/CD3 complex, along with co-stimulatory domains such as 4-1BB or CD28, to provide activation signals for CAR-T cells. The CAR genes were usually delivered into patient-derived T cells via lentiviral vectors. However, the random integration feature of lentiviral vectors poses a theoretical risk for insertional mutagenesis, which could potentially contribute to secondary CAR-positive malignancies. Importantly, extensive clinical analyses indicate that such events are exceedingly rare and reveal no definite evidence directly linking CAR gene insertion to oncogenesis, despite case reports. In contrast, conventional cancer therapies such as chemotherapy, radiotherapy, and hematopoietic stem-cell transplantation are well established contributors to secondary malignancies (39, 40). To mitigate this risk, the CRISPR-Cas9 system was employed to targeted insertion CAR gene with poly (A) sequences into the first exon of the *TRAC* gene, thereby replacing the endogenous TCRα constant region with the CAR (41). These CAR-positive T cells were expanded *in vitro*. Following infusion, CAR-T cells recognize cells expressing target antigen via their scFv, leading to activation and elimination of target cells through cytokine secretion (e.g., perforin and granzyme B) or engagement of apoptotic pathways such as factor associated suicide and its ligand (Fas/FasL) (42) (**Figure 1**).

Beyond oncology, the application of CAR-T therapy has recently expanded into the field of AIDs, where it shows great promise as a novel immunomodulatory approach (43). Given the shared characteristics between B-cell-driven malignancies and AIDs, such as B-cell hyperactivation and excessive autoantibody production, CAR-T-mediated B-cell depletion may offer therapeutic benefits (**Table 1**). For instance, patients with SLE often exhibit elevated frequencies of CD19^+^CD20^−^ B-cells, which are associated with autoantibody secretion (44). Moreover, animal studies have demonstrated that the infusion of anti-CD19 CAR-T cells effectively alleviates manifestations in murine SLE models (45).

**Table 1:** Published AIDs treatments by B-cell-targeting CAR-T cells.

Disease	Target	Patient number	CRS	Outcome	Ref.
SLE	CD19	5 patients	Grade1	Drug-free remission more than 8 months B-cell reconstitution within 110 days	(37)
SLE	CD19	1 patient	/	Profound improvement in both autoantibody titers and clinical disease activity scores	(33)
SSc	CD19	1 patient	Grade 1	Decreased autoantibody level Attenuated fibroblast activation	(34)
IIM	CD19	1 patient	Grade 1	Clinical-serological remission Myositis resolved Immunoglobulins restored	(36)
MG	BCMA	14 patients	/	Clinical improvement occurred despite continued immunosuppressive therapy and persistent autoantibodies/serum IgG	(63)
MS	CD19	2 patients	Grade 1	CAR-T cell enrichment in the CSF Reduced intrathecal antibodies	(35)
MG	CD19	1 patient	/	Clinical scores improved Reduced AChR	(43)
IMNM/SSc	CD19	3 patients	/	Complete B-cell depletion within 2 weeks; deep remission at 6 months; reversal of inflammation/fibrosis	(89)
SLE	BCM/CD19	12 patients	Grade 1	Reduced autoantibodies and prolonged disease	(101)
SLE with LN	BCM/CD19	10 patients	Grade 1	Complete clearance of all autoantibodies; B-cell reconstitution within 2–6 months	(10)

SLE, Systemic Lupus Erythematosus; SSc, Systemic Sclerosis; MG, Myasthenia Gravis; MS, Multiple Sclerosis; IIM, Idiopathic Inflammatory Myopathies; CRS, Cytokine Release Syndrome; ICANS, Immune Effector Cell-Associated Neurotoxicity Syndrome. IMNM, Immune-Mediated Necrotizing Myopathy; LN, lupus nephritis.

However, regarding the differences in the mechanisms of AIDs and tumor, CAR-T strategies should be carefully evaluated before clinical use. In cancer patients, CRS and immune effector cell-associated neurotoxicity syndrome (ICANS) are major safety concerns, both arising from excessive cytokine release (e.g., IFN-γ, IL-6) by activated CAR-T cells. In a compassionate use report from Fabian Müller et al., 15 patients with severe AIDs—including 8 with SLE, 3 with idiopathic inflammatory myositis, and 4 with systemic sclerosis—were treated with anti-CD19 CAR-T cells. The treatment induced profound B-cell depletion and achieved sustained drug-free remission in all patients, with only one in each disease group experienced manageable grade 2 CRS and ICANS (46). This suggests a favorable safety profile of CAR-T therapy in AIDs compared to oncology settings. In cancer therapy, lymphodepletion is considered essential to enhance the efficacy of infused CAR-T cells. This conditioning regimen eliminates immunosuppressive cells such as Tregs and myeloid-derived suppressor cells (MDSCs), thereby creating a favorable microenvironment for CAR-T cell expansion and function (47). It also increases the bioavailability of homeostatic cytokines like IL-7 and IL-15, which are critical for T cell persistence and antitumor activity (48). For example, lymphodepletion significantly improved the therapeutic efficacy of Tisagenlecleucel, a commercial anti-CD19 CAR-T product, in treating refractory diffuse large B-cell lymphoma, enhancing CAR-T cell expansion *in vivo*, prolonging patients’ progression-free survival, and increasing their remission rates (49).

**Figure 2:**
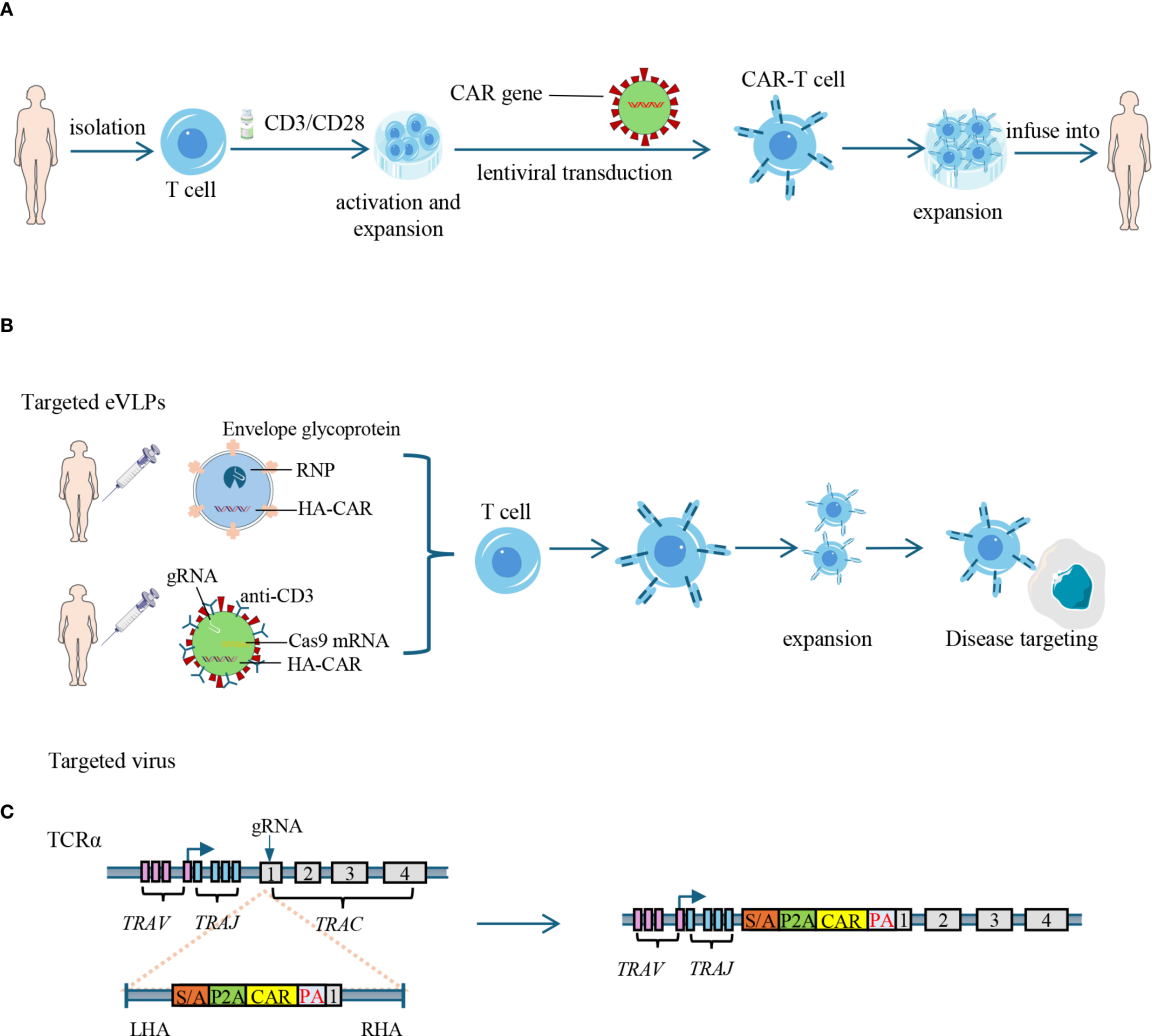
Strategies for CAR-T cell generation: *in vitro* engineering and *in vivo* delivery. **(A)** Ex vivo generation of CAR-T cells: T cells are isolated from peripheral blood and activated using CD3/CD28 stimulation. CAR genes are introduced via lentiviral transduction. The engineered CAR-T cells are then expanded *in vitro* and infused back into the patient. CAR: chimeric antigen receptor. **(B)** *In vivo* delivery of CAR constructs: engineered eVLPs carrying RNP (comprised Cas9 protein, crRNA and tracrRNA) and HA-CAR, or targeted virus carrying Cas9 mRNA, gRNAs, and HA-CAR are administered to directly transduce T cells *in vivo*, bypassing the need of lymphodepletion. eVLPs: engineered virus-like particles; RNP: ribonucleoprotein; HA-CAR: homology arms-flanking CAR sequence. **(C)** CRISPR-mediated targeted insertion of CAR into the *TRAC* locus: guide RNAs (gRNAs) and HA-CAR are used to disrupt the endogenous TCR and insert the CAR gene into the first exon of TCR α constant (*TRAC*) gene, reducing risks of graft-versus-host disease in allogeneic settings. RNP, ribonucleoprotein; *TRAV*, T cell receptor alpha variable region gene; *TRAJ*, T cell receptor alpha joining region gene; *TRAC*, T cell receptor alpha constant region gene; SA, splice acceptor; P2A, 2A peptide; PA, poly-A tail; LHA, left homology arm; RHA, right homology arm; eVLPs, Engineered virus-like particles.

Nevertheless, the role of lymphodepletion in AID treatment may differ from that in cancers, as the preservation of autologous immune cell subsets–particularly Tregs–is crucial for re-establishing immune homeostasis after CAR-T mediated depletion of pathogenic cells. Among available regimens, cyclophosphamide selectively depletes alloreactive T cells while sparing Tregs, facilitating the reconstitution of immune tolerance (50, 51). The combination of cyclophosphamide and fludarabine has proven effective in AIDs patients receiving anti-CD19 CAR-T cell therapy, with manageable adverse effects such as nausea, fatigue, and cytopenia (37, 46). However, the benefit-risk profile of cyclophosphamide requires carefully consideration—particularly in young female patients—due to its well-documented ovarian toxicity and the associated risk of infertility (52). To further obviate the need of lymphodepletion, *in vivo* CAR gene delivery approaches have been explored, such as target-specific lentiviral vectors that transduce T cells directly within the patients (53). These vectors introduce guide RNA (gRNA), Cas9 mRNA, and a CAR transgene flanked by homology arms (HA-CAR) into T cells. The targeting specificity is achieved through the display of anti-CD4 or anti-CD8 antibodies on the viral capsid (54). However, these approaches raise concerns regarding off-target transduction and limited control over CAR expression. To address these issues, engineered DNA-free virus-like particles (eVLPs) have emerged as promising vehicles, combining the key advantages of both viral and non-viral delivery. The eVLPs efficiently package and deliver macromolecules—such as base editor or Cas9 ribonucleoproteins (RNP, comprised Cas9 protein, crRNA and tracrRNA)—without integrating foreign DNA. Through the incorporation of pseudotyping glycoproteins with defined tropism, eVLPs allow for tissue- and cell-specific delivery, enabling precise, transient, and safe gene editing *in vivo* (55) (**Figure 2**).

Chronic B-cell aplasia is the most expected side effect in cancer patients treated with anti-CD19 CAR-T cells, often necessitate life-long immunoglobulin replacement therapy (IRT) to prevent infections (49). In contrast, B-cell aplasia in AIDs patients is typically transient, with B-cell counts recovering within a year—a median duration approximately 90 days—indicating different kinetics of CAR-T cells in the context of AIDs and malignancies (36). This difference was further confirmed by a recent comparative study by Muller et al, which demonstrated that CAR-T cell persistence in SLE patients (median: 110 days) is markedly shorter than that in B-cell lymphoma (median: 740 days). Likewise, immune reconstitution following CAR-T-induced B-cell depletion occurred significantly faster in SLE patients (155 days) compared to lymphoma patients (798 days) (4). These findings highlight the divergent behavior of CAR-T cells in malignant and autoimmune settings. Mechanistically, the continuous presence of CD19-expressing tumor cells provides sustained antigenic stimulation, driving prolonged CAR-T cell expansion and cytotoxic activity. This environment supports the generation of long-lived memory CAR-T cells—particularly CD45RO^+^CD27^+^CD8^+^ subsets—that can persist and rapidly regain effector function upon re-encounter with tumor antigens (56). Notably, tumor environment—particularly that in the bone marrow—provides a specialized survival niche with enriched cytokines such as IL-7 and IL-15, which are essential for the maintenance of hematopoietic stem cells and memory T cells (57). In contrast, CAR-T cells in AIDs primarily target autoreactive B-cells residing secondary lymphoid organs such as lymph nodes and spleen. These anatomical compartments lack the specialized stromal and cytokine-rich milieu characteristic of the bone marrow, and thus do not support the establishment of long-lived CAR-T cell memory (58). Importantly, the inflammatory environment in AIDs typically resolves quickly following therapy, marked by rapid declines in cytokines like IL-6 and CXCL13. Simultaneously, homeostatic cytokines such as TGF-β1, CXCL12, and IL-7 rebound, promoting endogenous lymphocyte reconstitution. However, these factors are insufficient to support the prolonged survival of CAR-T cells, ultimately leading to a loss of therapeutic persistence (4). In addition, the antigenic burden in AIDs is generally lower and more transient than in malignancies, leading to rapid clearance of target cells. This results in an abrupt reduction in antigenic stimulation and subsequent contraction of the CAR-T cell (59). Moreover, leukapheresis products from SLE patients typically contain higher proportions of naïve T cells, which give rise to CAR-T cell products enriched in central memory phenotypes at their peak. These central-memory CAR-T cells exhibit shorter *in vivo* persistence than effector-dominant populations seen in B-cell lymphoma (4). Furthermore, AIDs patients typically retain an intact immune system, including functional hematopoietic niches that facilitate B-cell recovery once CAR-T activity subsides (37). CAR-T cells-associated immunodeficiency is more reversible and tolerable in AIDs, largely due to a combination of less supportive tissue microenvironments, lower antigen burden, intrinsic characteristics of the CAR-T cell products, and preserved hematopoietic function—all of which collectively favor their clinical applicability. However, a recently identified, AID-specific adverse effect of anti-CD19 CAR-T cell therapy—termed local immune effector cell-associated toxicity syndrome (LICATS)—garnered attention due to its high incidence (30 out of 39 patients affected). LICATS manifestations are strictly confined to organs previously affected by the underlying autoimmune pathology—for instance, skin and kidneys in SLE, or muscle in myositis— with skin (19 events) and renal (12 events) involvement being the most frequent among 54 reported events. These manifestations typically emerge at a median of 10 days post-infusion, during the phase of B-cell aplasia. They are generally self-limited, with a median duration of 11 days, and predominantly mild in severity (Grade 1–2). Distinct from CRS—which typically manifests within one day post-infusion and is characterized by elevated IL-6 levels—LICATS presents with a delayed onset, exhibits organ-specific localization, and lacks systemic IL-6 elevation. In parallel, LICATS also differs from classical autoimmune flares by the absence of characteristic serologic markers and its limited responsiveness to conventional immunosuppressive therapy. Rather than indicating disease relapse, LICATS is more likely a localized inflammatory reaction triggered by CAR-T-mediated clearance of tissue-resident autoreactive B cells. Nevertheless, its underlying mechanisms remain to be elucidated in future studies (60).

## CAR-engineered T cells tailored to the characteristics of autoimmune diseases

**Figure 3:**
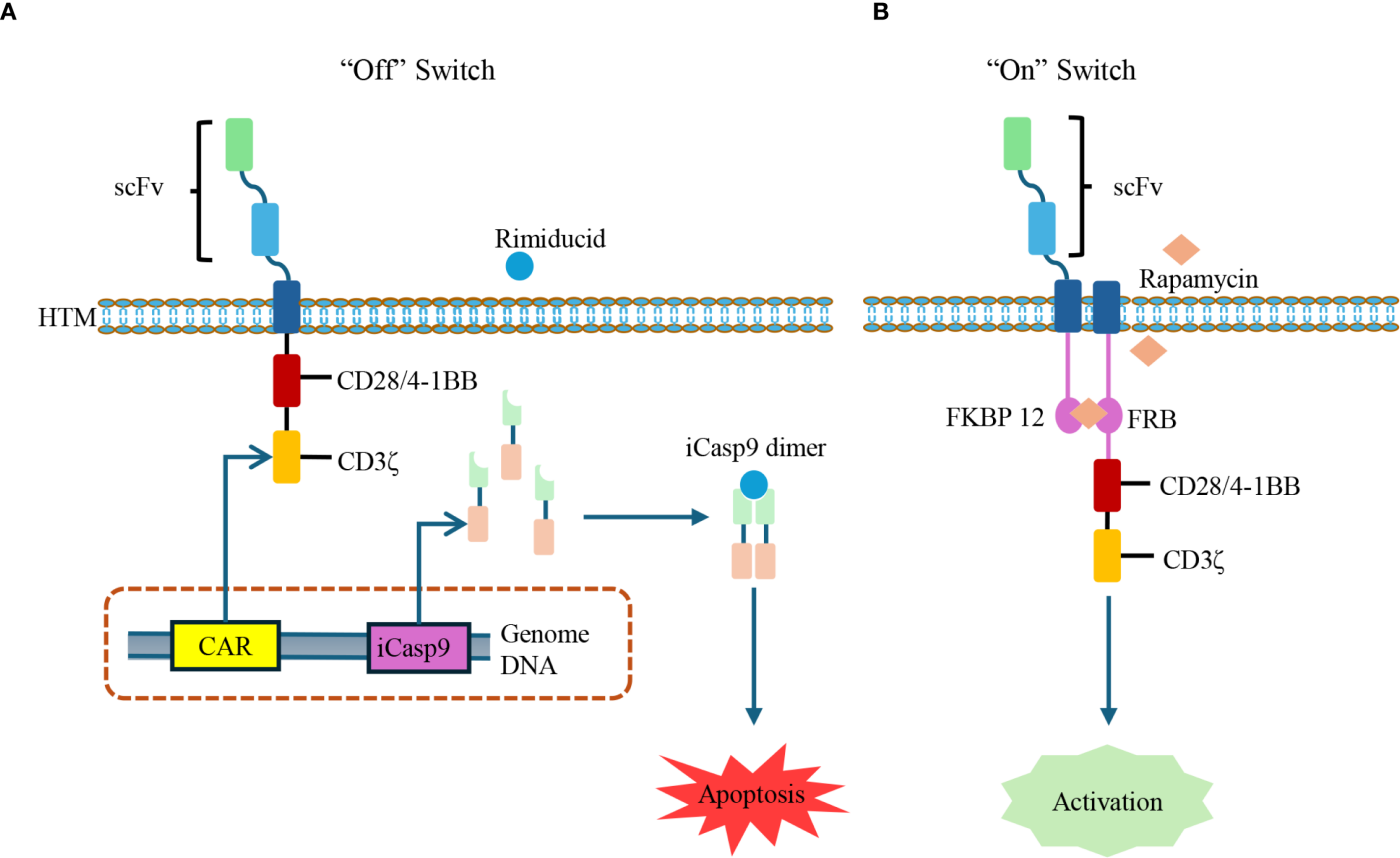
Controllable CAR-T cells with inducible safety switches. **(A)** Drug-inducible suicide switch: The iCasp-9 system is integrated into CAR-T cells. Upon administration of Rimiducid, iCasp-9 dimerizes and activates downstream caspase signaling, leading to CAR-T cell apoptosis and rapid termination of activity. iCasp-9: inducible caspase-9. **(B)** Drug-inducible activation switch: The CAR structure is split with FKBP12 and FRB domains, which can be dimerized in the presence of Rapamycin. This interaction restores CAR signaling, thereby enabling CAR-T cells activation in a ligand-dependent manner.

The applicability of CAR-T therapy needs to be carefully designed and evaluated to address the characteristics of AIDs. One prominent concern is the heightened risk of infection due to B-cell depletion, especially in patients previously exposed to immunosuppressive regimens. To mitigate potential safety issues, researchers have developed CAR constructs equipped with “safety switches” that allow for conditional control of CAR-T cell activation and persistence. For instance, inducible caspase-9 (iCasp9) functions as an “off switch”, triggering apoptosis of CAR-T cells upon administration of Rimiducid (61). In contrast, a drug-inducible “on switch” system has been designed using Rapamycin to dimerize FKBP-12 and FRB domains separately fused to the CAR scFv and intracellular signaling domains, thereby activating CAR-T cells only upon antigen binding and Rapamycin administration (62). These safety systems significantly enhance the controllability and safety of CAR-T therapy in non-malignant settings (**Figure 3**).

While long-term CAR expression is often essential in cancer therapy, time-dependent CAR expression has gained favor in the context of AIDs due to its improved safety profile. Transient CAR-T cells are generated by transfecting T cells with CAR-encoding mRNA, allowing temporary CAR expression and reducing the risk of prolonged off-target effects or cytokine storms. A recent clinical study in 14 patients with myasthenia gravis demonstrated that transient CAR-T cells induced clinical benefit with favorable safety and tolerability (63). Despite the advantages of transient CAR-T cells, challenges remain, including the inherent instability of mRNA and the notoriously low transfection efficiency of T cells by exogenous mRNA (64). Advances in nucleoside-modified mRNA technology have significantly improved mRNA stability and reduced activation of Toll-like receptors (TLRs), facilitating *in vivo* mRNA delivery (65). Furthermore, targeted lipid nanoparticles (tLNPs) have been engineered to specifically deliver this nucleoside-modified mRNA to T cells by surface-conjugated antibody, achieving effective and targeted gene expression in preclinical models (66, 67). These advances highlight the potential of tLNPs-mediated *in vivo* CAR-T generation as a promising non-viral approach for AIDs.

Unlike B-cell malignancies where CD19^+^ cells are abundant, the pathogenic B-cells in AIDs often represent a minor population expressing disease-specific B-cell receptors (BCRs). To selectively target these autoreactive B-cells, researchers have developed CAAR-T, in which the scFv is replaced with autoantigen epitopes (68). This modification enables “reverse targeting” of autoreactive B-cells that recognize these epitopes through their BCRs (69). Currently, Phase I clinical trials are evaluating CAAR-T therapies targeting mucosal pemphigoid (NCT04422912) and MuSK myasthenia gravis (NCT05451212). Additionally, CAAR-NK cells targeting La/SSB autoantigen-specific B-cells have been developed by incorporating lupus autoantigen sequences into NK-92MI cells, showing promise in preclinical models (70).

**Figure 4:**
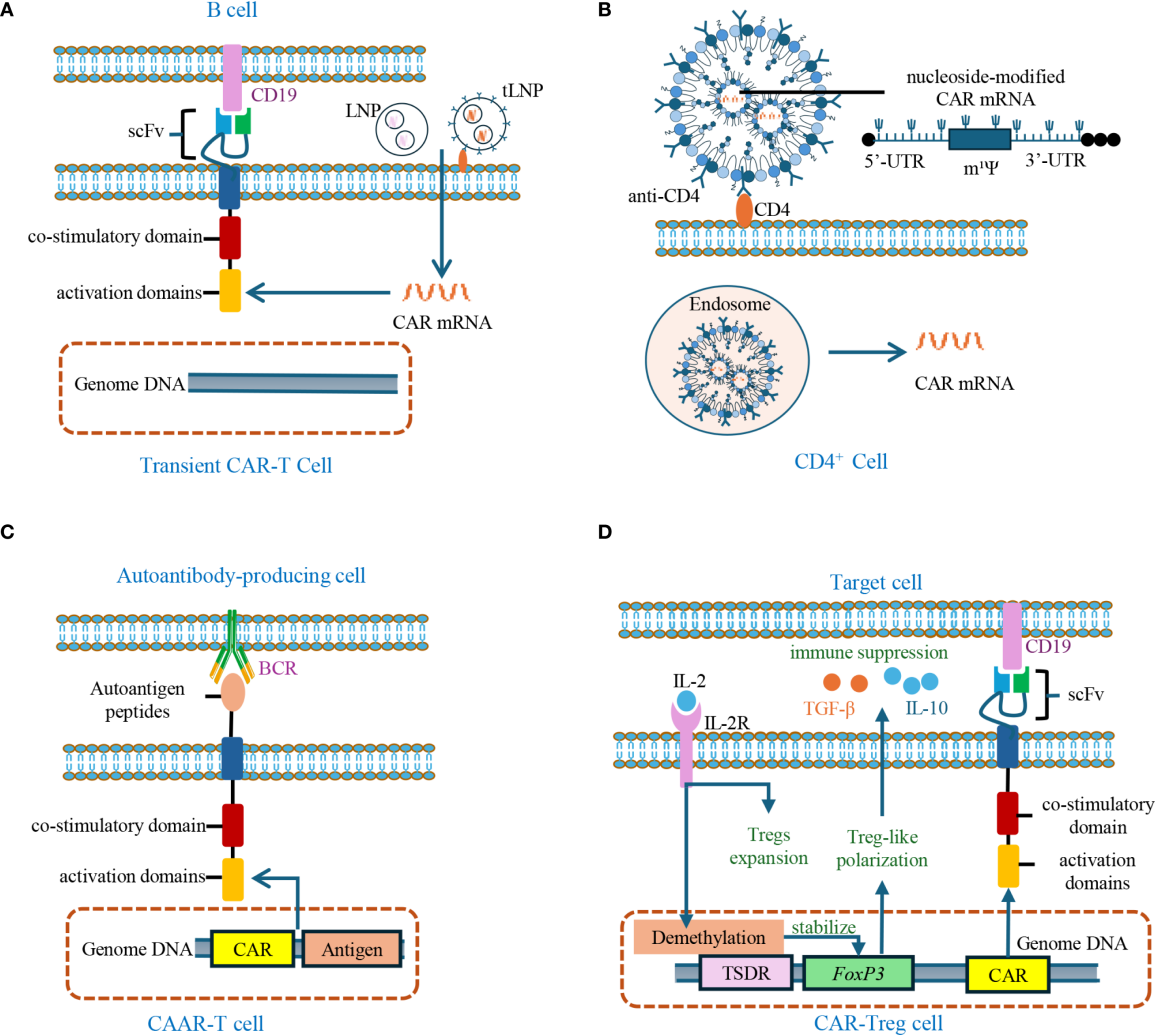
Engineered CAR-T cells and CAR-Tregs for autoimmune disease treatment. **(A)** Transient CAR-T cells are generated by delivering LNP or tLNP-encapsulated CAR mRNA targeting CD19 into T cells. Unlike conventional CAR-T cells, CAR expression here is transient and does not integrate into genomic DNA. LNP: lipid nanoparticle; tLNP: targeted lipid nanoparticle. **(B)** Schematic of tLNP-mediated CAR mRNA delivery: anti-CD4-targeted lipid nanoparticles encapsulating nucleoside-modified CAR mRNA (uridine replaced by 1-methylpseudouridine, m¹Ψ) are internalized by T cells through endocytosis, resulting in transient CAR expression. **(C)** CAAR-T cells are engineered to express autoantigen peptides in their extracellular domains, enabling selective targeting and depletion of autoantibody-producing cells via BCR engagement. CAAR: Chimeric autoantibody receptor; BCR: B-cell receptor. **(D)** CAR-Treg cells are engineered through *FoxP3* overexpression or stabilization via TSDR demethylation in CD4^+^ T cells expressing tissue-specific CAR, thereby inducing a Treg-like phenotype. Upon antigen recognition, these CAR-Treg cells exert immunosuppressive functions, primarily by secreting IL-10 and TGF-β. *FoxP3:* Forkhead box P3; TSDR: Treg-specific demethylated region; CAR: chimeric antigen receptor; IL-2R: interleukin-2 receptor.

Given the critical role of Tregs in restoring immune homeostasis, their therapeutic use in AIDs is gaining attention. However, their low abundance in circulation poses a barrier to clinical applications. To overcome this, researchers have engineered Tregs (EngTregs) by transducing *FoxP3* into CD4^+^ T cells (**Figure 4**). Buckner et al. pioneered an HDR-based gene editing approach to enforce stable FOXP3 expression in bulk CD4^+^ T cells, generating functional EngTregs with a durable phenotype and potent suppressive capacity (71). Beyond *FoxP3* overexpression, alternative strategies have emerged, including the *in vitro* induction of Tregs through IL-2 stimulation (72), *in vivo* expansion using low-dose IL-2 (73), and epigenetic modifications—such as inhibition of histone deacetylases and selective demethylation of the Treg-specific demethylated region (TSDR)—to stabilize *FoxP3* expression (74, 75). Recent findings also underscore the pivotal role of PI3Kδ signaling in Treg homeostasis, where gain-of-function mutations paradoxically impair suppressive activity despite expanding Treg numbers, positioning this pathway as a therapeutic target (76). For antigen-specific applications, TCR-modified EngTregs have been generated to recognize a novel PDC-E2 epitope in primary biliary cholangitis, allowing precise suppression of pathogenic T cell responses (77). To further enhance tissue specificity, these engineered Tregs have been equipped with antigen-specific receptors, giving rise to CAR-Tregs (78). Building on this concept, efforts are now directed toward optimizing CAR-Treg design and delivery. A representative advance is the dual HDR editing platform developed by the Rawlings group, which integrates *FOXP3* stabilization, TRAC-targeted CAR insertion, and a chemical-inducible IL-2 system to improve EngTreg persistence and function (79). Complementing these cellular approaches, advances in nanomedicine have facilitated next-generation Treg therapies. Nanoparticle-based delivery systems enable targeted *in vivo* expansion of antigen-specific Tregs by encapsulating Treg-promoting cytokines or autoantigens, thereby enhancing local immune tolerance without systemic immunosuppression (80). Upon homing to inflamed tissues, these CAR-Tregs exert local immunosuppressive effects through cytokine secretion (TGF-β and IL-10) and induction of apoptosis in effector cells, with minimal systemic immune disruption (81). CAR-Tregs have shown promise in the field of transplantation, with HLA-A2-specific CAR-Tregs currently under clinical evaluation in renal (NCT04817774) and hepatic (NCT05234190) allograft recipients. In the context of AIDs, insulin-specific CAR-Tregs exhibited prolonged *in vivo* persistence—remaining detectable for approximately four months in a mouse model of type 1 diabetes—but did not improve disease outcomes (82). Preliminary data indicate myelin oligodendrocyte glycoprotein (MOG)-CAR-Tregs secrete remyelination-promoting factors, positioning them as a potential therapeutic approach for multiple sclerosis (MS) where neuroprotection is paramount (83). In inflammatory autoimmune conditions, CAR-Tregs targeting the interleukin-23 receptor (IL-23R) effectively suppressed pathogenic Th17 cell responses and attenuated colitis in preclinical models of Crohn’s disease (CD) (84). In addition, flagellin derived from Escherichia coli H18 (FliC)-specific CAR-Tregs preferentially home to the inflamed colon, suppress pathogenic T cells, and promote epithelial barrier integrity in preclinical models of inflammatory bowel disease (IBD) (85). More recently, a preclinical study evaluated Fox19CAR-Tregs—Mengineered by overexpressing FoxP3 and harboring an anti-CD19 CAR—for the treatment of SLE. The encouraging results showed that a single infusion of Fox19CAR-Tregs suppressed autoantibody production, delayed lymphopenia, and restored immune homeostasis within lymphoid organs in a humanized mouse model, all without detectable toxicity. Despite a limited survival duration, Fox19CAR-Tregs effectively protected SLE-affected organs with high efficacy and safety, supporting further exploration of this therapeutic approach (86).

Nevertheless, the use of patient-derived autologous T cells for CAR-T manufacturing presents practical challenges, including prolonged production timelines and high commercial costs. These limitations have spurred interest in developing allogeneic, “off-the-shelf” CAR-T products to facilitate broader clinical application (87). Using CRISPR-Cas9, genes encoding HLA-A and HLA-B are knocked out from donor T cells to avoid host-versus-graft rejection (HvGR), while retention of HLA-C/E/G preserves NK cell tolerance (88). Additionally, deletion of the TCR α constant (*TRAC*) gene prevents graft-versus-host disease (GvHD) by eliminating native TCR expression (9). In a recent clinical trial, allogeneic anti-CD19 CAR-T cells with *PDCD1* gene knockout achieved sustained B-cell depletion and drug-free remission for over six months in patients with myositis and systemic sclerosis, with no severe adverse events (89). This marked the first clinical application of *PDCD1* knockout in allogeneic CAR-T therapy for AIDs, a strategy previously adopted in oncology to prolong CAR-T persistence. Moreover, a clinical trial evaluated allogeneic anti-CD19 CAR-T cells (TyU19), engineered using CRISPR/Cas9 to disrupt TRAC, HLA-A, HLA-B, CIITA, and PD-1 in patients with refractory SLE. This trial employed reduced-intensity lymphodepletion regimen that excluded anti-CD52 antibodies. TyU19 demonstrated robust expansion and persistence for over two months, with only grade 1 CRS observed. These results suggest that such genetic modifications effectively prevent immune rejection while preserving a favorable safety profile (90).

## Utilizing innate immune cells for CAR-based therapies in autoimmune diseases

In malignancies, CAR-T therapy faces several limitations inherent to its mechanism and technical requirements. These include poor tissue infiltration, susceptibility to exhaustion, complex immune regulatory environments, and excessive cytokine release upon activation (91). Additionally, CAR-T cells preparation demands high-quality homogeneous T cells and involves substantial manufacturing costs, limiting its broader clinical applicability (92).

To address these challenges, alternative immune cell types have been investigated as potential platforms for CAR engineering. NK cells are MHC-unrestricted cytotoxic immune cells that are capable of lysing target cells by secreting granzyme B and perforin. Unlike T cells, NK cells derived from healthy donors can be readily prepared into off-the-shelf CAR-NK products, thereby avoiding the dysfunction or immunosuppression often seen in AIDs, meanwhile shortening patients’ waiting times and significantly lower treatment costs (93). Additionally, as target antigen loss is a major cause of CAR-T therapy failure, CAR-NK activation relies on the recognition of natural receptors, thus reducing the likelihood of target cell immune escape. CAR-NK therapy also induces less inflammatory cytokine release during cytotoxic activity, mitigating the risk of adverse effects including CRS and neurotoxicity compared to CAR-T therapy (94). Encouraging results support this approach: a trial using cryopreserved allogeneic CAR-NK cells in B-cell malignancies reported an overall response rate of 80% (95). Furthermore, clinical trials are exploring the use of CD19-targeted CAR-NK cells in SLE (NCT06010472, NCT06421701) (**Table 2**).

**Table 2:** Clinical trials using CAR-based cells for AIDs therapy.

National clinical trial	Disease	Target	Cell platform	Phase
NCT06056921	SLE, SS, SSc, DM, ANCA	CD19	CAR-T	I
NCT06508346	ANCA	CD19	CAR-T	I
NCT06347718	SLE	CD19	CAR-T	Early I
NCT06222853	SLE	CD19	CAR-T	I
NCT06106906	SLE	CD19	CAR-T	I/II
NCT06691152	SLE	CD19	CAR-T	I
NCT05828212	NMO	CD19	CAR-T	I
NCT05828225	MG	CD19	CAR-T	I
NCT06019889	MG	CD19	CAR-T	II
NCT06384976	MS	CD19	CAR-T	I/II
NCT06342960	LN	CD19	CAR-T	I/II
NCT06121297	SLE, LN	CD19	CAR-T	I/II
NCT06428188	SLE	CD19/BCMA	Dual-specific CAR-T	I
NCT05858684	SLE	CD19/BCMA	Dual-specific CAR-T	I
NCT05085431	SS	CD19/BCMA	Dual-specific CAR-T	Early I
NCT05085444	Scleroderma	CD19/BCMA	Dual-specific CAR-T	Early I
NCT05085418	IN, LN	CD19/BCMA	Dual-specific CAR-T	Early I
NCT06350110	SLE, LN	CD19/BCMA	Dual-specific CAR-T	I
NCT05263817	POEMS, AL	CD19/BCMA	Dual-specific CAR-T	Early I
NCT05474885	R/R SLE	CD19/BCMA	Dual-specific CAR-T	I
NCT05846347	SLE	CD19/BCMA	Dual-specific CAR-T	I
NCT04422912	Mucosal PV	DSG3	CAAR-T	I
NCT05451212	MuSK MG	MuSK	CAAR-T	I
NCT06421701	SLE	CD19	CAR-NK	I
NCT06010472	SLE	CD19	CAR- NK (KN5501)	Early I

SLE, Systemic Lupus Erythematosus; SS, Sjögren’s Syndrome; SSc, Systemic Sclerosis; DM, Dermatomyositis; ANCA, Antineutrophil Cytoplasmic Antibody; NMO, Neuromyelitis Optica; MG, Myasthenia Gravis; IN, Immune Nephritis; POEMS, Polyneuropathy, Organomegaly, Endocrinopathy, Monoclonal Protein, Skin changes; AL, Amyloid Light Chain; R/R SLE, Relapsed/Refractory Systemic Lupus Erythematosus; Mucosal PV, Mucosal-Dominant Pemphigus Vulgaris; MuSK MG, MuSK Myasthenia Gravis; MS, Multiple Sclerosis.

Macrophages have also emerged as another promising cell platform for CAR-based therapies, particularly for addressing the limited infiltration and immunosuppressive environments in solid tissues (96). In oncology, HER2-specific CAR-macrophages have demonstrated both robust tumor infiltration and anti-tumor activity in HER2-positive tumors, highlighting their potential utility in non-systemic AIDs that affect solid organs (97). Moreover, macrophages also play roles in tissue repair. In rheumatoid arthritis (RA), a reduction in TREM2^+^ tissue-resident macrophages is often observed, impairing phagocytic clearance and bone homeostasis, thereby exacerbating disease progression (98). These observations underscore the therapeutic potential of macrophages in modulating immune responses and promoting tissue repair in AIDs.

## Synthetic biology expands the potential to CAR-based therapies for autoimmune diseases

Synthetic biology, a transformative discipline that emerged in the 21st century, focuses on constructing programmable biological circuits using fundamental biological elements such as DNA and genes. Its central objective is to engineer “artificial cells” that can sense their environment and respond to specific stimuli. This rapidly evolving field holds significant promise for advancing CAR-based cell therapies.

For instance, although anti-CD19 CAR-T cells have demonstrated remarkable efficacy in depleting B-cells, their utility in AIDs is limited by two main issues: off-target effects—since CD19 is not expressed on autoantibody-producing plasma cells, and the potential downregulation or loss of CD19 on B-cells after repeated interactions with anti-CD19 CAR-T cells (99). To address these challenges, researchers have drawn inspiration from “logic gates” in synthetic biology to achieve precise control over CAR-T cell activation (100). By engineering CAR-T cells to recognize multiple antigens—including CD19, CD20, and the plasma cell marker BCMA—via shared or independent intracellular signaling domains, these cells can be activated upon encountering any of the target antigens (101). This approach, known as an “OR gate,” expands antigen recognition and enhances therapeutic efficacy.

**Figure 5:**
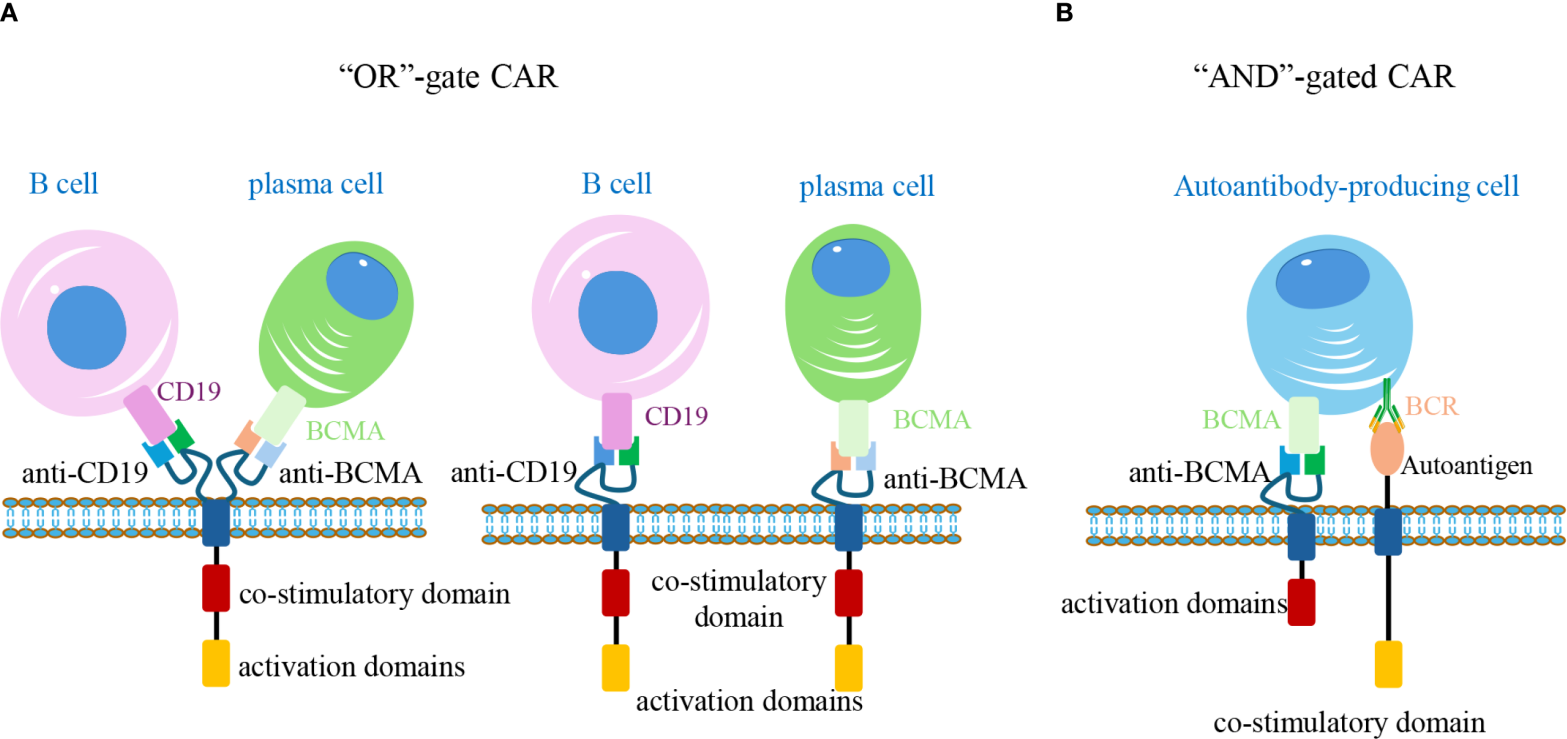
Logic-gated CAR-T cells for precise targeting of autoreactive cells. **(A)** “OR” gate design: CAR-T cells express tandem CARs recognizing CD19 and BCMA. Binding to either antigen (on B-cells or plasma cells, respectively) is sufficient to activate the cell, expanding the therapeutic coverage. **(B)** “AND” gate design: Two separate CARs are engineered with distinct intracellular signaling domains. Full activation occurs only when both BCMA and autoantigen-specific BCR are engaged on the same target cell, enabling selective elimination of pathogenic autoantibody-producing cells while sparing non-pathogenic counterparts. CAR, chimeric antigen receptor; BCR, B-cell receptor.

Another advanced design in CAR therapies involves the implementation of “AND gates” logic (88). Similar to rapamycin—inducible safety switches, AND gates assign intracellular signaling domains (e.g., CD3, 4-1BB, or CD28) downstream to distinct scFvs. Activation occurs only when all CARs simultaneously bind their target antigens, ensuring that only cells expressing all target markers trigger a full response. This design minimizes “on-target/off-cell” toxicity and enhances therapeutic precision by sparing healthy tissues that may share single antigens with pathogenic cells (102). For example, anti-CD19/BCMA bispecific CAR-T cells have been developed to selectively eliminate autoantibody-producing cells, allowing for targeted immune modulation while preserving broader immune function (103). Currently, seven ongoing Phase I/II clinical trials are evaluating anti-CD19/BCMA dual-targeting CAR-T in refractory AIDs (**Figure 5**).

Beyond logic gates, synthetic biology has also enabled the development of advanced artificial signaling systems, notably the synthetic Notch (SynNotch) platform. Modeled after endogenous Notch pathway, SynNotch system utilizes a mechanically activated extracellular domain—such as an scFv binding to an autoantigen—to initiate a proteolytic cascade. This results in the release of natural or artificial transcription factors, which subsequently drive the expression of specific gene or secondary regulators (104).

**Figure 6:**
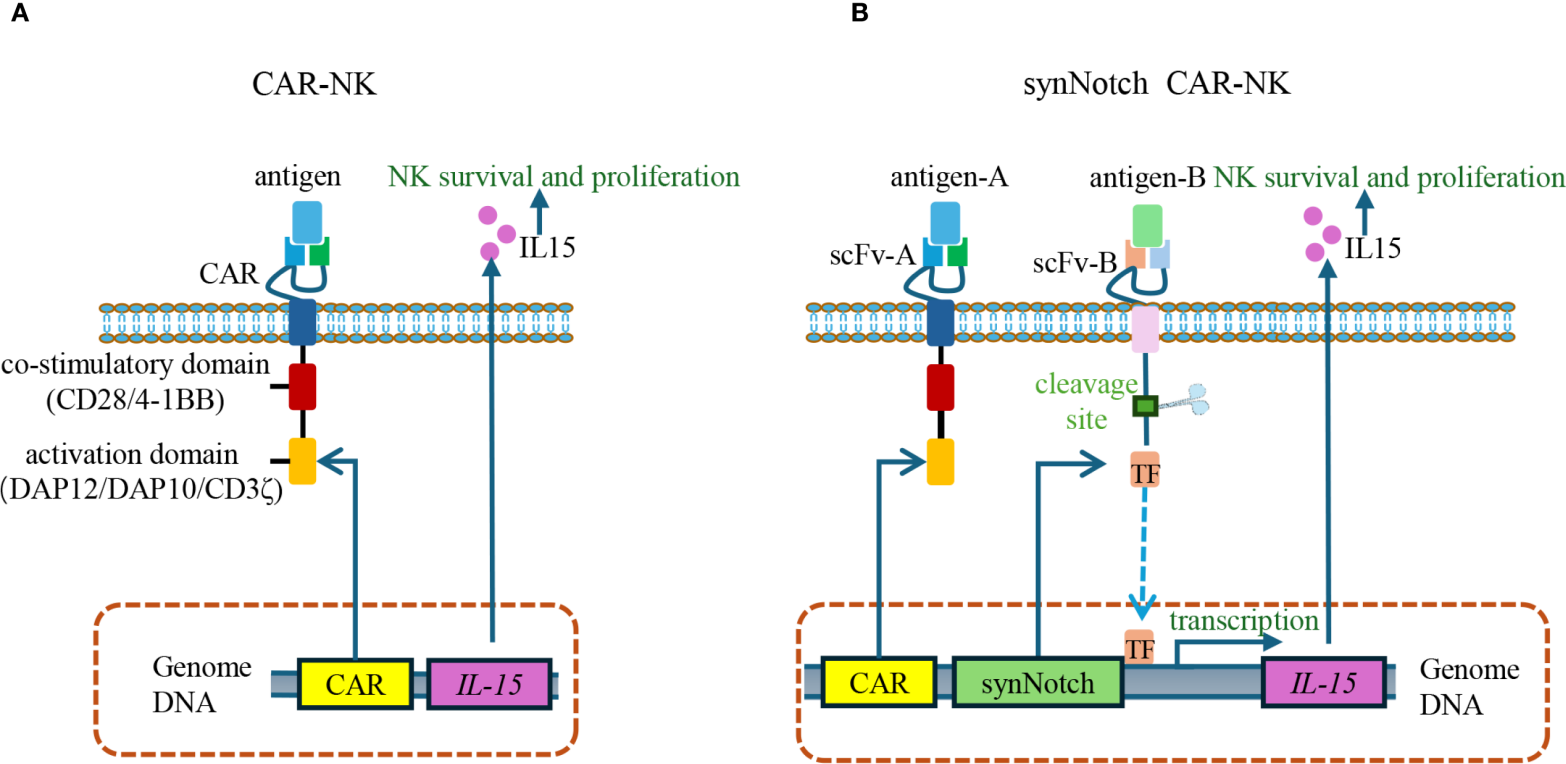
SynNotch-regulated CAR-NK cells for conditional IL-15 expression. **(A)** Conventional CAR-NK cells constitutively express IL-15 to promote NK cell survival, proliferation, and cytotoxic function. However, continuous IL-15 expression can induce off-target toxicity due to the undesired activation of host NK cells. **(B)** SynNotch CAR-NK cells use scFv-A to recognize antigen A and mediate cytotoxicity, while scFv-B detects antigen B to activate the SynNotch pathway. Upon antigen B binding, a transcription factor is released to induce IL-15 expression. Once antigen B is no longer present, IL-15 production stops, thereby enhancing safety by limiting cytokine release to dual-antigen recognition. scFv, single-chain variable fragment.

For instance, CAR-NK cells, while offering certain advantages over CAR-T cells, face challenges due to their short lifespan—typically less than 10 days—which necessitates frequent infusions to maintain sufficient cell number (105). To address this limitation, researchers have incorporated interleukin-15 (IL-15) mRNA into CAR constructs, given IL-15’s role in enhancing the proliferation, persistence, and homing capacity of CAR-NK cells. However, constitutive IL-15 expression also increases the risk of non-specific activation of host NK cells (106). The integration of SynNotch technology has provided an effective solution: upon recognition and binding of the CAR-NK scFv to its target antigen, the SynNotch signaling pathway is activated, thereby inducing the expression and release of IL-15 (107). Once target antigen engagement ceases, SynNotch signaling halts, leading to rapid IL-15 withdrawal and subsequent CAR-NK cell apoptosis. This dynamic regulation enhances both the specificity and safety of the therapy (108) (**Figure 6**).

## Conclusion and further thinkings

CAR-based cell therapy, which has demonstrated remarkable success in treating malignancies, has also made significant strides in the management of AIDs. Despite encouraging preclinical and early clinical findings, several challenges must be addressed to ensure its safe and effective application in the autoimmune setting.

First, AIDs patients often present with compromised immune cells due to long-term immunosuppressive treatment. Moreover, unlike in cancer, lymphodepletion may not be desirable in AIDs treatment, as preserving endogenous immune cells is essential for restoring immune homeostasis and preventing long-term immunodeficiency. Additionally, CAR-T cell therapy can induce acute B-cell aplasia, thereby increasing the risk of infections.

To overcome these limitations, researchers have developed refined CAR construction equipped with safety switches, and use mRNA-based transient CAR expression, enhancing the controllability of CAR activation and minimizing adverse effects. Therapeutic specificity has also been optimized using chimeric autoantibody receptor T (CAAR-T) cells and multi-antigen targeting CARs, such as CD19/BCMA bispecific constructs, to eliminate pathogenic B-cells while sparing normal immune components. Furthermore, CAR-Tregs have emerged as a strategy for restoring immune tolerance by delivering regulatory signals directly to inflamed tissues, thus achieving therapeutic effects with minimal systemic disruption.

In parallel, novel immune cell types such as NK cells and macrophages have been investigated as alternative platforms for CAR engineering. CAR-NK cells offer advantages including MHC-independent killing, reduced cytokine release, and the potential for “off-the-shelf” manufacturing, with promising results in early-stage trials. CAR-macrophages, on the other hand, demonstrate superior tissue infiltration and remodeling capacity, making them particularly attractive for targeting localized, organ-specific AIDs.

Synthetic biology has further expanded the potential of CAR-based therapies by introducing programmable logic and dynamic control systems. Logic gate-based CAR constructions allow for more precise discrimination of pathological cells, while the SynNotch signaling pathways enable context-dependent activation of therapeutic functions, such as inducible IL-15 production in CAR-NK cells (**Table 3**).

**Table 3:** Engineered CAR-immune cells in AIDs therapies.

CAR-engineered cells	Advantages	Key technology	Limitations	Stage of development
Allogeneic CAR-T	“Off-the-shelf” availability, faster access; avoids lengthy autologous manufacturing	CRISPR-mediated targeted insertion of CAR into the *TRAC* locus	Risk of graft rejection/GvHD	Clinical trials ongoing (Phase I/II)
With Safety switches	Drug-inducible control over CAR-T function	iCasp9-induced apoptosis, Split-CAR-mediated activation	Requires administration of exogenous drugs	Preclinical validation; early clinical evaluation
*in vivo* CAR-T	Targeted *in vivo* CAR-gene delivery	Anti-CD3 or anti-CD4 antibody modification on LNP surfaces	Limited control over CAR expression	Preclinical development
With Nucleoside modified mRNA	Enhances mRNA stability and reduces innate immune activation	Replacement of uridine with m¹Ψ	Instability of mRNA	Conceptual Design
CAAR-T	Selective clearance of autoreactive B-cells	Autoepitope to engage pathogenic BCRs	limited to BCR-expressing cells	Phase I clinical trials ongoing
CAR-Tregs	Immune tolerance reconstitution	CAR-T cells engineered to stably express FoxP3	Complex manufacturing	Preclinical validation; early clinical trials in transplantation
CAR-NK	Lower CRS/neurotoxicity risk and MHC-independent cytotoxicity	Use of NK cells for CAR expression	Short persistence	Early Phase I clinical trials in SLE initiated
CAR-macrophage	Improved infiltration into inflamed tissues or solid lesions	Use of macrophages for CAR expression	Limited clinical data in AIDs	Preclinical development
“OR”-gated CAR cells	Broad antigen coverage	CARs recognizing either of two antigens for activation	Potential on-target/off-cell toxicity	Phase I/II clinical trials ongoing in refractory AIDs
“AND”-gated CAR cells	Reduced off-target toxicity	CAR activation requiring dual-antigen co-recognition	Reduced sensitivity; may miss low-antigen-expressing cells	Preclinical development
SynNotch CAR-NK	Context-specific activation and improved persistence	SynNotch-triggered IL-15 expression upon antigen encounter	Engineering complexity; untested in humans	Conceptual Design

Moving forward, the successful clinical translation of CAR-engineered therapies for AIDs hinges on overcoming key challenges such as antigen escape, immune tolerance restoration, and scalability. Rigorous validation of safety, durability, and immunological outcomes is essential. Critical clinical design considerations include defining therapeutic windows, optimizing preconditioning regimens, and developing standardized trial protocols to account for disease heterogeneity. Ultimately, interdisciplinary collaboration across immunology, bioengineering, and clinical medicine will be vital to translate these therapies into effective treatments for AIDs.
